# Anomalous Discharge Behavior of Graphite Nanosheet Electrodes in Lithium-Oxygen Batteries

**DOI:** 10.3390/ma13010043

**Published:** 2019-12-20

**Authors:** Philipp Wunderlich, Jannis Küpper, Ulrich Simon

**Affiliations:** Institute of Inorganic Chemistry, RWTH Aachen University, 52072 Aachen, Germany; jannis.kuepper@ac.rwth-aachen.de (J.K.); ulrich.simon@ac.rwth-aachen.de (U.S.)

**Keywords:** Li-O_2_, lithium-oxygen batteries, air electrode, graphene, nanographite, exfoliation

## Abstract

Lithium-oxygen (Li-O_2_) batteries require rational air electrode concepts to achieve high energy densities. We report a simple but effective electrode design based on graphite nanosheets (GNS) as active material to facilitate the discharge reaction. In contrast to other carbon forms we tested, GNS show a distinctive two-step discharge behavior. Fundamental aspects of the battery’s discharge profile were examined in different depths of discharge using scanning electron microscopy and electrochemical impedance spectroscopy. We attribute the second stage of discharge to the electrochemically induced expansion of graphite, which allows an increase in the discharge product uptake. Raman spectroscopy and powder X-ray diffraction confirmed the main discharge product to be Li_2_O_2_, which was found as particulate coating on GNS at the electrode top, and in damaged areas at the bottom together with Li_2_CO_3_ and Li_2_O. Large discharge capacity comes at a price: the chemical and structural integrity of the cathode suffers from graphite expansion and unwanted byproducts. In addition to the known instability of the electrode–electrolyte interface, new challenges emerge from high depths of discharge. The mechanistic origin of the observed effects, as well as air electrode design strategies to deal with them, are discussed in this study.

## 1. Introduction

After years of research without the anticipated breakthrough, the lithium-oxygen (Li-O_2_) battery is still a highly desired but elusive electrochemical system. The promise of high specific energy is tied to numerous fundamental challenges associated with a conversion-type cell chemistry, which need to be mastered in order to make it into application as an energy-storage system for e.g., long-range electric vehicles. With one solid and one gaseous reactant, Li-O_2_ cells comprise features of both lithium batteries and fuel cells, and require novel, highly adapted electrode designs. The use of a lithium metal electrode, which offers a lucrative specific capacity of 3862 mAh/g, is a challenge in itself [1]. Coupled with oxygen, its potential can be harnessed in an electrochemical reaction forming lithium peroxide [2]:

2 Li + O_2_ ⇌ Li_2_O_2_ (E^o^ = 2.96 V), (1)

The more oxygen is bound in the gas diffusion electrode during operation, the heavier the cell becomes. The amount of charge that is stored in the form of lithium peroxide (1168 mAh/g), multiplied with the equilibrium cell potential, gives an outstanding theoretical specific energy of 3458 Wh/kg. Including the weight of the cell stack, typically consisting of a lithium metal anode, a separator, an electrolyte and the air electrode, and peripheral components such as current collectors, an O_2_ tank or gas separation membranes, the specific energy was estimated to reach up to 500 Wh/kg – 1000 Wh/kg [3,4]. The advantage over the predominant Li-ion battery in terms of gravimetric energy storage capability is still a factor of two to three. As of 2019, Li-O_2_ batteries are far from practical application, which is being attributed to their poor cycling stability, limited power density and low round-trip efficiency. There is a strong need to prevent electrolyte [5] and cathode [6] degradation. Highly reactive intermediates such as lithium superoxide (LiO_2_) or recently debated singlet oxygen are causes for parasitic side reactions that reduce the lifetime of a battery [7,8]. Strategies towards higher reversibility, such as the application of redox mediators [9] or the elimination of aprotic solvents by using solid-state [10] or molten salt electrolytes [2,11], have shown remarkable improvements in reversibility and cycle life.

In order to reach the full technological potential of the Li-O_2_ electrochemistry, batteries must be able to provide the highest possible energy density without tradeoffs for other properties. High capacities will be a practical requirement that comes with additional fundamental challenges. Air electrode materials that perform well under shallow discharge conditions may suffer from volume changes, electrolyte displacement, pore clogging or heavy dielectric passivation caused by the discharge products, if the electrode is fully discharged [12,13,14]. The deep discharge effects must also be explored and discussed in the context of cathode or carbon decomposition [15].

In terms of discharge performance, nanocarbons are the cathode materials of choice due to their electrochemical stability, conductivity and unique microstructure that allows the assembly of porous electrodes [16]. Carbon nanotubes (CNT) and graphene are the most prominent examples for lightweight electrode materials that enable remarkable specific capacities in Li-O_2_ batteries [17,18,19,20]. One way towards porous graphene electrodes is the employment of graphene nanosheets [21,22,23]. Still, graphite is an appealing Li-O_2_ cathode material from a technological perspective, because it is fairly abundant, low-cost and well-researched [24,25,26,27]. Compared to graphene, graphite electrodes are rarely reported, because the formation of discharge products takes place on the cathode surface and graphene-like materials mainly offer more specific surface area than milled graphite.

In this work, we chose graphite nanosheets (GNS) as a cathode nanomaterial that closes the gap between graphene and graphite. We loaded the nanosheets into a polymer foam substrate to create a gas diffusion electrode that provides sufficient open pore space for discharge products. This electrode is a stable basis on which we build our electrochemical experiments to uncover the unusual deep discharge behavior of Li-O_2_ batteries with GNS cathodes. The discharge voltage profile is discussed and the cell passivation is probed in capacity-limited discharge experiments in combination with electrochemical impedance spectroscopy (EIS). The discharge products are analyzed by means of scanning electron microscopy (SEM), energy-dispersive X-ray spectroscopy (EDS), Raman spectroscopy and X-ray diffraction (XRD).

## 2. Materials and Methods

For GNS-loaded foam electrodes, commercially available melamine foam was cut into slices of about 1.5 mm thickness and 18 mm diameter discs were cut out using a custom-built tool. In order to turn them into current collector substrates, samples were sputter-coated with gold from both sides (100 W DC argon plasma at 10^−2^ mbar for 165 s) depositing 1.8 mg Au on average on each foam disc. The conductive foam was submerged in a dispersion of 40 mg GNS (Strem Chemicals, Inc., Newburyport, MA, United States) in 1.0 mL isopropanol under sonication for 10 min, removing any excess liquid not held by the scaffold. Samples were dried at 100 °C in glass vials overnight. Typical GNS-foam electrodes with a 5 mg substrate weighed 15 ± 2 mg.

Li-O_2_ cells were assembled in an argon-filled glovebox (<0.1 ppm O_2_ and H_2_O) using a modified version of the “ECC-Air” cell design from EL-Cell GmbH. All materials were dried at 100 °C overnight prior to use, except for the solvents, which were dried with 4 Å molecular sieves. Electrolytes were obtained by mixing tetraethylene glycol dimethyl ether (TEGDME, >99%, Sigma-Aldrich, Steinheim, Germany,) with LiNO_3_ (battery grade 99.999%, Alfa Aesar, Karlsruhe, Germany) for a 0.5 M solution. A lithium disc (18 mm × 300 µm, >99.8%, Rockwood, Frankfurt am Main, Germany) was covered with a 20 mm polymer-ceramic sheet separator (20 µm in thickness, FS-3025, Freudenberg, Weinheim, Germany) and homogeneously wetted with 200 µL electrolyte after the cathode was inserted. The cell was sealed, removed from the glovebox and connected to the O_2_ line with an inlet-type check valve that allowed the cell to be flushed with O_2_ (99.999%, <3 ppm H_2_O, Westfalen AG, Münster, Germany). The oxygen gauge pressure was 3 bar.

The Li-O_2_ cells were allowed to rest for 6 h before any electrochemical test. Galvanostatic measurements were carried out using a two-electrode setup connected to a Basytec CTS Lab (Asselfingen, Germany) battery tester. The standard current density of 150 µA/cm^2^ was normalized to the geometrical cathode surface area (2.54 cm^2^). All voltages were referred to the electrode potential of Li/Li^+^. Discharge tests were terminated as soon as the cell potentials fell below 2.0 V. EIS measurements were performed using a Zahner IM6 potentiostat and were measured with 10 steps per decade in a frequency range from 100 kHz to 10 mHz and 13 mV amplitude around the equilibrium cell potential. Data integrity was checked with the Kramers–Kronig relation.

Post mortem cathode characterization was performed by opening the discharged cells in a glovebox, removing the cathodes from the stack and carefully washing them with 1 mL 1,2-dimethoxyethane (DME, >99.5%, Sigma-Aldrich) in order to remove residual electrolyte, without changing the cathode structure. SEM images were taken with a LEO Supra 35 VP from Carl Zeiss AG (Oberkochen, Germany) using an in-lens SE detector and an acceleration voltage of 5 kV. Minor air exposure of several seconds while transferring the samples from an argon-filled transport container into the SEM chamber did not affect the discharge product morphology. Raman spectra were recorded with a LamRAM 300 from Horiba (*λ* = 633 nm, *p* = 12 mW, grating: 1800, magnification: ×500, grid: 7 cm^−1^) combined with an Olympus (Tokyo, Japan) BX-41 microscope. Measurements took approximately 2 min per spectrum and all spectra were baseline-corrected. Reference spectra of lithium compounds were recorded with the following commercial substances: Li_2_CO_3_ (Sigma-Aldrich, 100%), Li_2_O_2_ (Alfa Aesar, 95%), Li_2_O (Alfa Aesar, 99.5%), LiOH (Alfa Aesar, 98%) and the aforementioned LiNO_3_. XRD was carried out with a StadiP powder diffractometer by Stoe&Cie with Cu Kα (1.54 Å) radiation. The electrode pieces were sealed in a sample holder using acetate foil for the measurement duration of 2 h. Reference data for Li_2_O_2_, graphite and gold were taken from the Inorganic Crystal Structure Database (ICSD).

## 3. Results and Discussion

### 3.1. Electrode Microstructure

**Figure 1:**
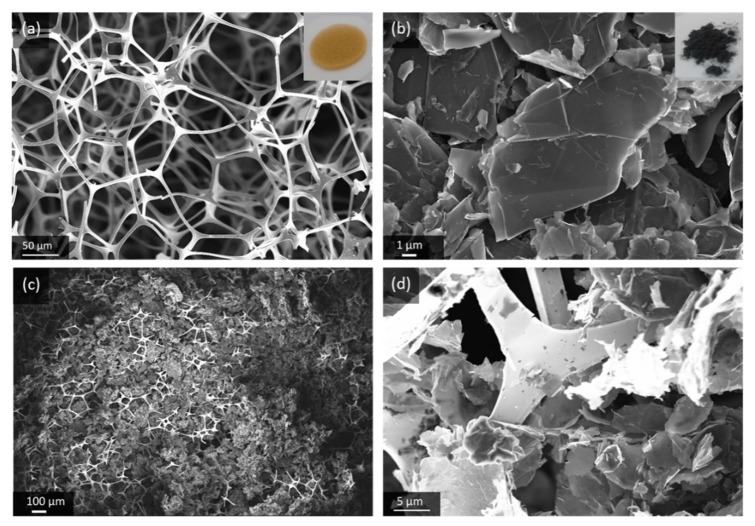
Scanning electron microscope (SEM) images of (**a**) Au-coated melamine foam, (**b**) pristine graphite nanosheets (GNS) and (**c**,**d**) GNS-loaded foam electrode at different magnifications.

GNS are highly crystalline carbon with only few structural and chemical defects, and a distinct anisotropic morphology that makes them suitable building blocks for porous microarchitectures. The material used in this work is an exfoliated graphite powder with sheet thickness of 5–10 nm and a lateral dimension of 25 µm. The Brunauer-Emmett-Teller (BET) surface area of 84 m^2^/g, which we measured, is closer to mildly processed natural graphite (7 m^2^/g) [27] than highly exfoliated graphene-like carbon (up to 2630 m^2^/g) [28]. The low bulk density of GNS, compared to conventional graphite powder, is visualized in Figure S1. In order to create a highly porous gas diffusion electrode, the GNS are loaded into a suitable substrate, which here is an Au-coated melamine foam. The polymer foam disc is flexible and with only 2 mg/cm^2^ it is lighter than other common substrates for gas diffusion electrodes such as nickel foams (≈ 40 mg/cm^2^) or carbon fibers (4 mg/cm^2^–10 mg/cm^2^). The composite microstructure of the GNS-loaded Au-coated melamine foam electrode (GNS-foam) is shown in Figure 1.

The electrode processing, by dip-coating in GNS-isopropanol dispersion, results in a typical electrode mass of 15 ± 2 mg. The GNS make up for 66% of the electrode with mass loading of around 3.9 mg/cm^2^. The electrode is designed to have mainly macropores, because meso- and micropores are more prone to clogging by discharge products [29,30]. The effective porosity of GNS-loaded foams in fully assembled battery cells is difficult to quantify, because the stack is compressed by a spring (about 69 kPa mechanical pressure on the cell stack), which reduces the cathode thickness to about 1 mm. The use of binders is avoided due to their chemical instability in Li-O_2_ batteries and because their decomposition products drastically increase the complexity of the cell chemistry [31,32]. On the one hand, individual GNS are, therefore, not fixed in place and they can rearrange to compensate for volume changes, but on the other hand, this increases the risk of out-of-contact particles. For the investigation of the deep discharge behavior of GNS as cathode material, the simplicity of the electrode components outweighs the need for a mechanically more resilient electrode.

### 3.2. Discharge Profile

**Figure 2:**
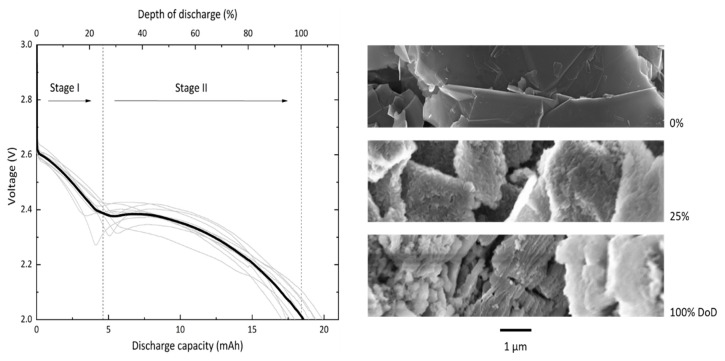
Ten individual discharge profiles (grey lines) and average profile (black line) of GNS-foams discharged at 150 µA/cm^2^. The discharge profile is divided in two stages that last to about 25% depth of discharge (DoD) and 100% DoD, respectively. The SEM images show representative GNS in their pristine state (0 mAh), and after the first (5 mAh) and second stage of discharge (18.4 mAh).

Discharge experiments are carried out at a current density of 150 µA/cm^2^ with a standard cell consisting of a GNS-loaded foam filled with 200 µL of 0.5 M LiNO_3_-TEGDME as electrolyte. In this study, the absolute values for cell capacities are reported and all necessary information for conversions are provided. The voltage profiles of 10 GNS-foam cells and representative SEM images of GNS with and without discharge products are shown in Figure 2.

The GNS-foam standard cell delivers a discharge capacity of 18.4 mAh ± 1.0 mAh (1654 mAh/g_carbon_ ± 473 mAh/g_carbon_), which is now set as 100% depth of discharge (DoD). The specific discharge capacity of GNS is competitive with high-surface-area carbon nanomaterials (>1000 mAh/g_carbon_) [6,16,17]. The relative capacity deviation of 5.6% may be caused by fluctuating temperatures in the laboratory, varying GNS-loadings, electrolyte volumes and also by randomly distributed pores and particles. This is an acceptable value for Li-O_2_ cells with individually processed electrodes [33,34]. The background capacity contribution of the Au-coated foam is measured under the same conditions except for no GNS were loaded into the substrate. The Au-coated melamine foam itself shows only negligible activity with a discharge capacity of 38 µAh. Discharging GNS-foam cells without O_2_ gives a similar background capacity of 37 µAh. Thus, for normal GNS-foams, the discharge capacity originates from electrochemical reactions that involve the GNS and O_2_.

The most striking characteristic of the discharge profile is a reproducible two-step feature. We define the first stage of discharge by its voltage range from the initial cell equilibrium potential down to about 2.4 V, and the second stage of discharge from this point to the voltage cut-off at 2.0 V. Cells typically show a monotonously falling voltage, which in some cases is not strictly valid due to a voltage dip at the stage transition. To rule out that the two-step profile is caused by a systematic error such as water contamination, additional tests were carried out with two non-graphite carbon electrodes (Figure S2). Cells built with commercial CNT or carbon black electrodes only show the expected discharge behavior of a Li-O_2_ battery, which is a monotonous voltage decay followed by a sharp potential drop when approaching the full discharge capacity [34,35,36]. Since no parameters other than the cathode were changed, we hypothesize that the two-step feature is in fact related to the GNS.

The SEM images in Figure 2 show pristine GNS next to those that are covered in discharge products as found in cathode samples of batteries that were discharged to 5 mAh (approx. 25% DoD) or 18.4 mAh (100% DoD). The sequence illustrates the structural evolution of the carbon particles through both stages of the discharge process: after stage I, discharge products cover the outer surface of individual GNS; after stage II, the particles are structurally decomposed. The process in the second stage of discharge likely includes the volume expansion of graphite segments, splaying of the graphene layers and continued deposition of discharge products. The discharge products in fully discharged GNS electrodes will be analyzed and discussed in detail in a later section.

### 3.3. Cell Passivation

**Figure 3:**
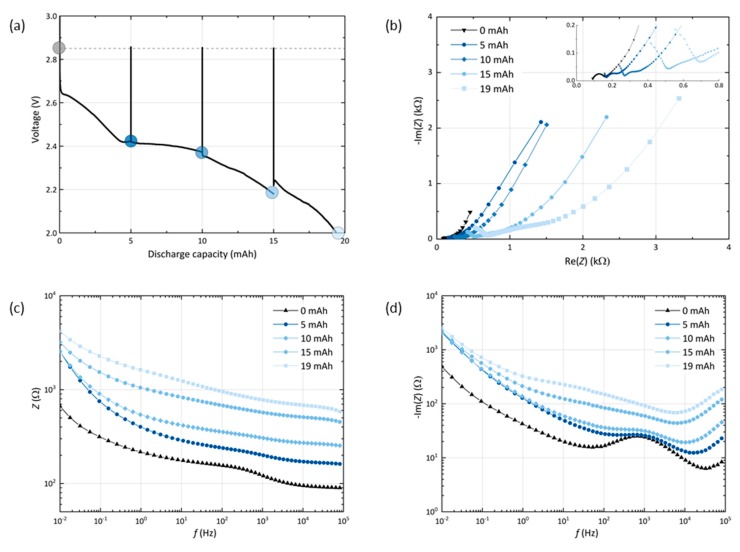
(**a**) Discharge profile of a GNS-foam cell with interruptions for the electrochemical impedance spectroscopy (EIS) after every 5 mAh (marked with dots). Resting steps ensure that the cell reaches its equilibrium potential before each measurement. (**b**) Nyquist, (**c**) Bode and (**d**) Bode-like plot with the impedance data of the cell.

To probe the cell passivation with respect to the depth of discharge, a GNS-foam cell is characterized by potentiostatic EIS measurements at equilibrium potential in its pristine state at 0 mAh and after every 5 mAh of discharge, which corresponds to approx. 25% DoD. Figure 3 shows the discharge curve with interruptions caused by EIS measurements (a) and the related impedance plots (b–d).

First, it is noted that the EIS measurements including all resting steps do not show significant impact on the discharge capacity (19.4 mAh), which is within the standard deviation range of the GNS-foams. The cell voltage recovers to 2.85 V after each spectrum measured. This value deviates from the theoretical value of 2.96 V, the potential of the cell reaction forming Li_2_O_2_, but is in good agreement with other experimental equilibrium voltages for comparable Li-O_2_ cells [15,35,37,38]. As expected, an increased depth of discharge leads to an increase of the cell impedance due to the deposition of insulating discharge products in the cathode [35]. The Nyquist plot (b) can be used to discuss the more fundamental contributions to cell impedance. The cell in its pristine state shows the commonly observed semicircle in combination with a low-frequency (LF) tail [35,39,40,41]. Equivalent circuit modelling of porous electrodes is complex and the assignment of certain geometries to fundamental processes is often uncertain. Therefore, one possible model is provided in the supplementary information (Figure S3) and we withhold from quantitatively describing the Nyquist plots.

In the Nyquist representation of the cell at 0 mAh, we assign the semicircle to the lithium anode and the LF slope to the porous GNS cathode, based on the impedance data that are obtained from symmetrical cells (Li-Li or GNS-GNS), which are depicted in Figure S4. When the cell is discharged, the semicircle is shifted to higher values and its diameter is increased, which indicates an increase in serial contact resistance and interfacial resistance. The end of discharge is typically reached when charge carrier transport through the discharge products film comes to a halt [14,35,42]. Depending on the morphology, porosity or crystallinity of the product film, the critical film thickness can range from a few to several hundred nanometers [14]. For GNS cells after discharge stage I (see EIS data point at 5 mAh), the charge transfer resistance of a nearly saturated particle surface is expected to rise sharply, however the cell has not reached its state of maximum passivation yet.

The Bode plot (c) indicates that the level of cell passivation keeps rising with each additional 5 mAh step in stage II, which means that the electrochemical reactions increase the amount of insulating discharge products. Yet, the highest impedance gains at the LF end occur during the initial 5 mAh step in stage I. In the Bode-like plot (d), it can be seen that the overall reactance levels rise above the initial values of the cell at 0 mAh, in which the lithium-related reactance around 1 kHz stands out. At first, the lithium-electrolyte interface dominates the mid-frequency reactance, but the more discharge product accumulates in the cathode, the larger the interfacial charge transfer resistance grows at the carbon-Li_2_O_2_-electrolyte interface. A measure of the electrochemically active surface area of the electrode is its double layer capacitance, which is inversely proportional to the reactance [43]. As the LF range is assigned to processes that are related to the porous cathode, the reactance values at the frequency of 10 mHz can be used as a parameter that allows the discussion of the surface coverage of the GNS electrode. The biggest loss of capacitance occurs after the first 25% DoD, while the other 75% do not further increase the LF reactance levels. This is in agreement with the processes we propose for the respective discharge steps: first, the outer GNS particle surface is covered and the remaining surface area reaches a minimum, then, the discharge reaction is sustained by the inner surface of GNS becoming accessible. Once the outer GNS surface is saturated and inaccessible for any further deposition of discharge products, it becomes feasible to facilitate reactions at this harder to reach interface. As the released surface becomes passivated immediately by new discharge products, the reactance values stay close to their maximum.

We anticipate that the rising level of GNS passivation caused by their full coverage with dielectric discharge product reduces the electrochemically available surface area in the electrode and increases the local current density in the remaining areas, thus resulting in the incrementally increasing overpotential. Only after stage II discharge, the battery reaches the point in which the cell impedance results in an overpotential that shifts the discharge voltage below the cut-off level. The increase in cell impedance by intrinsic passivation is and will remain one of the limiting parameters of cathode performance in Li-O_2_ batteries for the foreseeable future. Parts of the electrode become inaccessible if conductive paths become blocked, and subsequently removing the insulating materials will result in high charge overpotentials. Unlike H_2_-O_2_ fuel cells, where H_2_O as discharge product is removed from the air electrode, lithium oxides are bound to the electrode surface and, therefore, individual carbon particles can only be used *once* to generate capacity. In order to prevent dielectric passivation, the electronic properties of the discharge products need to be targeted, or cathodes should be greatly oversized. In the latter case, a defined amount of discharge product is distributed over a larger surface area, which in turn is not entirely passivated. This, however, will greatly lower the energy density of the Li-O_2_ battery.

### 3.4. Discharge Products

**Figure 4:**
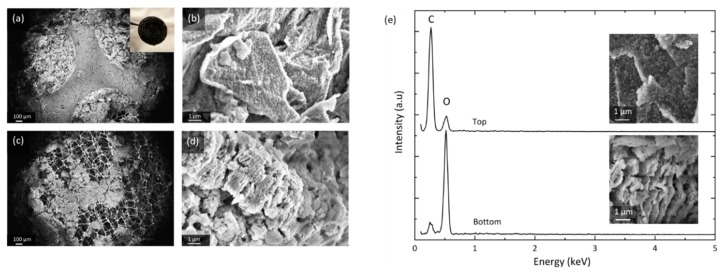
SEM of a GNS-foam electrode discharged to 18.9 mAh. (**a**) Overview of the electrode top side and (**b**) GNS coated with discharge products. (**c**) Overview of electrode bottom and (**d**) GNS that have undergone structural decomposition. (**e**) Energy-dispersive X-ray spectroscopy (EDS) spot spectra of GNS at electrode top and bottom with corresponding SEM images.

The characterization of discharged cathodes is carried out using post mortem SEM and EDS. Figure 4 shows the spatial distribution of GNS and discharge products at the top and bottom of a fully discharged GNS-foam.

The SEM images depicted in Figure 4 were taken in different areas of the same electrode and they show the heterogeneous distribution of reaction products. The top (a) can be divided into the areas covered and compressed by the current collector disc of the test cell hardware, and the neighboring circular areas that have direct access to the O_2_ headspace. GNS in the O_2_-rich zones (b) are fully encased in discharge products, whereas GNS in neighboring regions are textured and only partially coated (Figure S5a). The electrode bottom (c) shows severely degraded GNS: nanosheet stacks burst and greatly increase in volume (d), which macroscopically turns the electrode disc lumpy, brittle and greyish-white in color. After stage I discharge, more discharge products are found at the top of the electrode (at the O_2_-electrolyte interface) than at the bottom (the lithium-electrolyte interface), which is in accordance with most experimental reports [19,44,45,46]. After the full discharge, however, the bottom appears to be the more active area, where vast deposits of insulating discharge products lead to severe structural GNS degradation.

EDS data (e) suggest that the investigated GNS samples contain no elements other than carbon and oxygen after discharge. Note that lithium Kα_1_ radiation is not detected by most conventional EDS setups. It is safe to assume that the particles formed contain chemically bound oxygen. We can confirm the commonly observed decomposition of the discharge product particles when they are exposed to the electron beam [47]. This sensitivity is not observed in the heavily damaged and exfoliated GNS covered by insulating products. This is a possible indication that the discharge products locally have undergone side reactions that turn Li_2_O_2_ into more stable compounds (e.g., Li_2_CO_3_ or Li_2_O). The EDS spot measurements show a large carbon-to-oxygen signal ratio for GNS from the top (b) that only have thin particle coatings, whereas the ratio is inverted for the heavily degraded GNS from the bottom of the cathode (d). Since the O_2_ concentration is higher at the electrode top, but more O_2_ is chemically bound at the bottom, the O_2_ transport through the thick electrode seems to be sufficient and is not a limiting factor. A lack of Li^+^ transport could explain why there is less deep discharge activity at the cathode top, which is further away from the lithium anode.

**Figure 5:**
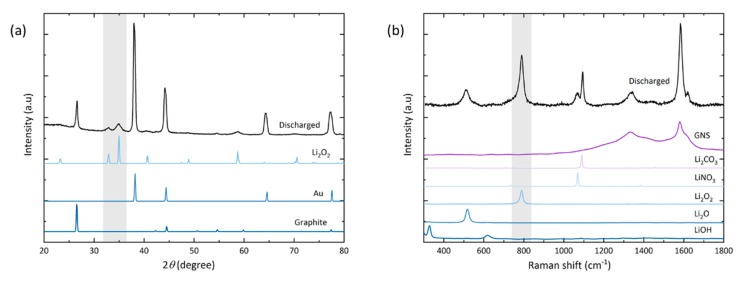
(**a**) X-ray diffractogram and (**b**) Raman spectrum of GNS electrode discharged to 18.9 mAh. The highlighted grey areas mark the peaks and Raman band of Li_2_O_2_, respectively.

Lastly, qualitative analysis of the main and side reaction products is carried out with X-ray diffraction and Raman spectroscopy (Figure 5).

In the diffractogram of the discharged electrode (a), peaks are observed for gold (as the coating of the foam substrate) and graphite (from the GNS). After full discharge, the peaks found at 2*θ* = 32.9° (001), 35.0° (101) and 58.7° (110) confirm the presence of crystalline Li_2_O_2_ as the main discharge product. No peaks of the typical side reaction products Li_2_CO_3_, LiOH or Li_2_O are observed. Their amounts may lie below the threshold of detectability in the measured sample volume, or these products are simply non-crystalline.

Some side reaction products are detected locally by Raman spectroscopy (b). The spot size of the laser is about 20 µm in diameter, which is in the same order of magnitude as the lateral dimension of the nanosheets. At the electrode top, no discharge product-related signals besides the graphitic carbon bands can be detected; likely due to the small amount of discharge products coupled with a low quantum yield of the Stokes shift. Hence our setup also did not allow us to characterize discharge products after stage I discharge. Signals of lithium compounds [48] are found on the electrode bottom with spot measurements that were carried out on greyish-white particles, which correspond to the heavily exfoliated GNS (Figure 4d). Besides the GNS G-band (1580 cm^−1^), the peroxide peak at 790 cm^−1^ is the most intense signal followed by Li_2_CO_3_ (1095 cm^−1^). Li_2_CO_3_ appears as a common side reaction product [49]. By the absence of LiOH signals, we assume that there was only negligible water contamination with no negative effect. We attempted to minimize any exposure to air by operating the Raman setup under constant argon flow and carefully monitoring possible discolorations of a lithium disc next to the sample. The Li_2_CO_3_ signal is detected in the deteriorated parts of the electrode bottom and it is reasonable to assume that it stems from GNS decomposition. Yet, due to the known general instability of the electrolyte, it is also reasonable to assume that it was partially caused by degradation of TEGDME [8,50,51]. Residues of LiNO_3_ (1085 cm^−1^) as the electrolyte salt are also detected. Another experimentally observed effect is that the Li_2_O_2_ signal diminishes if measurements are repeated on the same spot (Figure S6). The Li_2_CO_3_ signal is less affected by this and, just like an electron beam, the Raman laser promotes thermal decomposition of the product to be analyzed. Another band at around 516 cm^−1^ is found in the discharged sample, matching the commercial Li_2_O powder. This signal is only found on structurally damaged GNS next to the Raman bands of Li_2_O_2_ and Li_2_CO_3_.

The predominant discharge product even in the highly degraded regions of the electrode is still Li_2_O_2_ and with respect to the discharge profile, we assume that it is deposited during the first stage of discharge and then undergoes further reaction to Li_2_O and Li_2_CO_3_. Two possible routes are either the chemical reaction of the metastable Li_2_O_2_ with carbon or a consecutive electrochemical reduction step. The chemical route has been discussed by McCloskey et al. and may occur via the following reactions [50,51]:

2 Li_2_O_2_ + C → Li_2_O + Li_2_CO_3_, (2)

Li_2_O_2_ + C + 0.5 O_2_ → Li_2_CO_3_, (3)

This reaction is self-limiting and stops after a few monolayers of Li_2_CO_3_ have been formed at the carbon–Li_2_O_2_ interface [50]. However, for exfoliated GNS, where graphene becomes accessible, the newly gained interface can promote these side reactions. In terms of carbon stability, highly crystalline graphite generally proves to be superior to disordered carbons due to lower defect concentrations, which could promote electrolyte and cathode degradation [52,53]. Considering the capacity-voltage profile of the GNS cells, the side reactions could be faradaic and the formation of Li_2_O then involves another two-electron reduction step [2]:

Li_2_O_2_ + 2 Li^+^ + 2 e^−^ → 2 Li_2_O, (4)

Without overcoming thermodynamic and kinetic barriers, Li_2_O_2_ is the more stable discharge product, but Li_2_O can be obtained at high temperatures using a suitable catalyst [2]. Furthermore, LiNO_3_ as electrolyte salt can trigger a parasitic reaction pathway that involves the formation of intermediate LiNO_2_ and turns Li_2_O_2_ into Li_2_O and Li_2_CO_3_ [2]. In our experiments, the Raman band of Li_2_O is found at ambient temperature, but at high DoD. Cells that show Li-O_2_-typical sudden death behavior at the end of discharge, only generate a small fraction of discharge capacity (<5% DoD) below a critical threshold and the amount of Li_2_O that is formed should be insignificant. In contrast to that, about 75% of the discharge capacity in the GNS-based system is obtained in discharge stage II in the voltage range between 2.35 V and 2.0 V (at 150 µA/cm^2^), in which Li_2_O could be formed. Other studies suggest that low potential discharge promotes the formation of byproducts like Li_2_CO_3_ or Li_2_O, which are one reason for the heavy passivation that leads to the poor performance in full capacity cycling experiments [37,38]. Since the second stage of discharge of GNS electrodes is a high-overpotential (inefficient) process, the structural damage and some side reaction products could be avoided by raising the cut-off voltage above 2.5 V, although it would greatly reduce the discharge capacity that can be reached with the material.

The two-step discharge profile may be the transition from an *exo*- to an *endoparticulate* mechanism. So far, graphite exfoliation in the context of Li-O_2_ batteries has been observed by Hirshberg et al., but only after extended cycling and the underlying mechanisms are unclear [54]. Here we observe that structural damage in graphite particles already occurs during discharge. The appearance of the two-step feature of the discharge profile is likely favored by our experimental setup that features a highly porous foam electrode and a moderate discharge current density. The deep discharge effects include graphite volume expansion, splaying of the graphene layers and even exfoliation that is coupled to the deposition of discharge products. How could the mechanism behind this unusual discharge behavior proceed?

Beyond the Li-O_2_ cell chemistry it is known (and technologically applied) that graphite is an excellent intercalation host for lithium ions. An unwanted effect in batteries is the cointercalation of solvent molecules, which in Li-ion batteries is solely prevented by a stable solid-electrolyte interphase (SEI) layer that passivates and stabilizes the graphite surface [55]. In other scientific contexts, the cointercalation is utilized to provoke graphene exfoliation e.g., in order to obtain materials like reduced graphene oxide (rGO) or graphene nanosheets [56]. Exfoliation can proceed in the following steps: (i) weakening of the van der Waals forces between the graphene layers, (ii) induction of mechanical stress and (iii) deformation that results in expansion and splaying of the layers [57]. In Li-O_2_ batteries, the cathode-electrolyte interface is far from stable and during discharge an expansionary product formation takes place. Volume changes are inevitable—even in highly porous electrodes. The observed degradation of GNS at the electrode bottom is a microscale symptom occurring only in combination with a certain set of discharge parameters and only in the presence of O_2_. The starting point of exfoliation could be graphite particles that are in contact with the electrolyte, which contains Li^+^, the salt anions (here: NO_3_^−^) and O_2_. Together, this may form a more or less stable SEI [58]. Solvent, O_2_ or ion (Li^+^, O_2_^−^) cointercalation via diffusion may be already be possible at this point. Starting at the edges of a graphene stack as the preferred sites for discharge product formation, individual layers are splayed to further expose their inner surface area [59]. Layers end up not only being expanded, but also immediately coated with compounds that are likely a mix of discharge products including Li_2_O_2_, Li_2_O and Li_2_CO_3_. Other signs of the structural degradation of GNS are fragmentation, rupture and decay into irregularly shaped particles (Figure S6). The more GNS that participate in this process, the higher will be the loss of active (conductive) material, which will increase the cell impedance. This supports the progressing passivation (Figure 3) in the second stage of discharge that occurs in addition to the growth of the Li_2_O_2_ particle film on top of the GNS. To reveal the mechanisms that prevail at a certain stage of discharge, the oxygen-electron consumption ratio must be quantified and coupled with analysis of the solid products [60]. Without suitable techniques, the exact mechanisms of GNS degradation and Li_2_O formation remain unclear, but local imbalances of the Li^+^ or O_2_ concentration and the local depth of discharge (e.g., the state of passivation of individual nanosheets) will certainly have an impact. Spatial variation in the electrical field, electrochemical potential and especially O_2_ and Li^+^ accessibility prevents a gradient-free cell from being built, especially since the reaction interface changes depending on the state of discharge. Adapted electrode microstructure and pore design could help to attenuate these effects.

The graphite expansion has not exclusively negative consequences, because GNS are theoretically capable of reaching a multiple of the discharge capacity they would reach, if only their outer particle surface provides space for discharge products. The electrochemically induced expansion and exfoliation of graphite could be exploited as a facile and inexpensive way to unlock the full discharge capability of graphite [56,57,61,62,63]. Presumably, the anomalous discharge behavior of GNS has implications for the deep discharge of any graphite- or multilayer graphene-based cathode, or even electrodes that employ other two-dimensional layered materials. The structural decomposition of the GNS is likely an irreversible effect, which may limit the rechargeability of the battery. This should be assessed systematically in cycling experiments, in which the cycled capacity is linked to various depths of discharge. Special attention should be paid to the choice of electrolyte, because it is known to strongly interfere with the discharge mechanism. Battery test parameters such as the current density, temperature, or the oxygen pressure should also be subject to future studies that elucidate circumstances under which the second stage of discharge is promoted.

## 4. Conclusions

In summary, Li-O_2_ batteries with graphite nanosheet-based electrodes showed a distinctive two-step discharge profile, which was attributed to the layered structure of the cathode material. This allowed a relatively low-surface-area material like GNS to reach capacities that are typically obtained with high-surface-area nanocarbons only. In the first stage, which is the conventional discharge behavior, Li_2_O_2_ is deposited on the surface of graphite particles until surface saturation. The second stage is a high-overpotential electrochemical process that requires the inner surface of GNS to be accessible in order to maintain the discharge current density. SEM images and EIS measurements confirmed the continued deposition of discharge products and structural degradation in conjunction with the related increase in cell impedance. Under deep discharge conditions, GNS underwent severe volume expansion in an irreversible manner. Raman spectroscopy and XRD showed that next to Li_2_O_2_, the byproducts Li_2_CO_3_ and Li_2_O_2_ are formed. Based on these findings it is recommended that Li-O_2_ batteries with graphite-based cathodes should not be discharged fully in order to minimize such unintended structural effects. Better understanding is necessary on what triggers the two-step profile and how this can either be prevented to avoid structural carbon decomposition, or harnessed for high-capacity Li-O_2_ batteries. Gaining full control over the electrochemistry behind this endoparticulate mechanism is a chance for simple graphite to become a high-performance electrode material for Li-O_2_ cells, which is the subject of our current research.

## Supplementary Materials

The following are available online at https://www.mdpi.com/1996-1944/13/1/43/s1: Additional SEM images, Raman spectra, EDS and electrochemical data.
