# Supplementary material for: Anomalous Discharge Behavior of Graphite Nanosheet Electrodes in Lithium-Oxygen Batteries
Source: Materials (Basel). 2019 Dec 20;13(1):43. doi: 10.3390/ma13010043 (PMC6982280; doi:10.3390/ma13010043)
Supplement: Supplementary file 1 [file materials-13-00043-s001.pdf]

materials

Supplementary Materials

Anomalous Discharge Behavior of Graphite Nanosheet Electrodes in Lithium-Oxygen Batteries

Philipp Wunderlich *, Jannis Küpper and Ulrich Simon

Institute of Inorganic Chemistry, RWTH Aachen University, 52072 Aachen, Germany; jannis.kuepper@ac.rwth-aachen.de (J.K.); ulrich.simon@ac.rwth-aachen.de (U.S.)

* Correspondence: philipp.wunderlich@ac.rwth-aachen.de; Tel.: +49-241-80-94644

Received: 28 November 2019; Accepted: 17 December 2019; Published: 20 December 2019

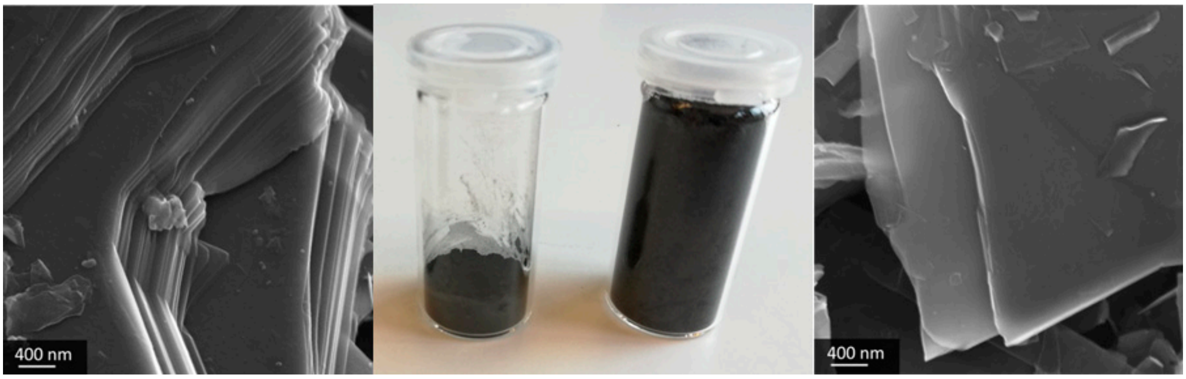

Figure S1. 500 mg each of graphite (Merck, mesh 325) and GNS (from Strem Chemicals, Inc., 25 μm) in direct comparison. The GNS in the right vial and SEM image demonstrate a significantly lower powder packing density than the less exfoliated graphite.

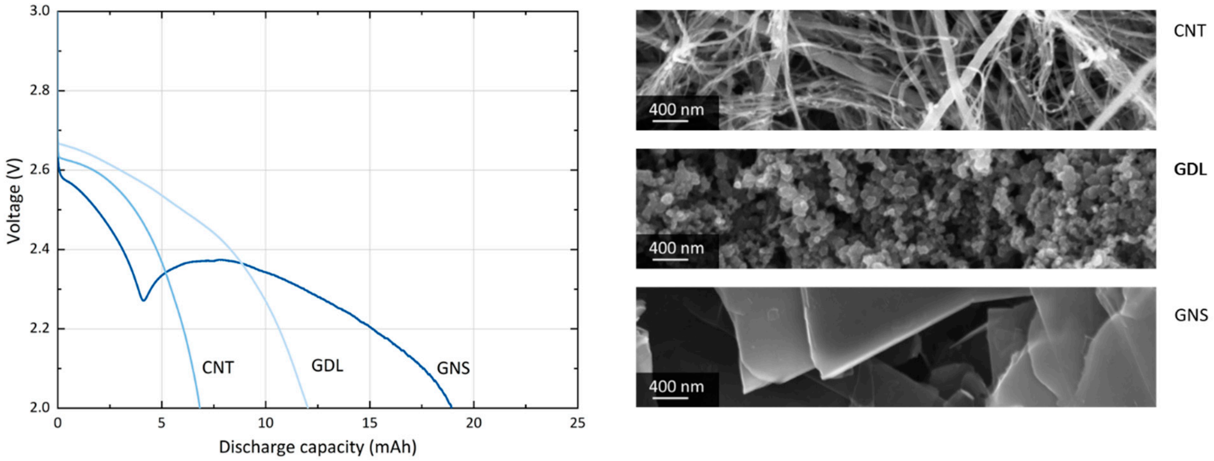

Figure S2. $U(Q)$ profiles of a GNS-loaded foam (13.7 mg) and two other carbon electrodes as references: a carbon nanotube (CNT) buckypaper, 4.8 mg (NanotechsLab) and a Super P-coated gas diffusion layer (GDL) electrode, 25.0 mg (GDL24BC, Sigracet). All cells are discharged at 150 μA/cm² with 200 μL of 0.5 M $LiNO_3$ in TEGDME as electrolyte. The SEM images show the pristine carbon materials before the battery testing.

Impedance model

The model used to describe the results is based on a modified, simplified transmission line model (TLM) that has been established and explained comprehensibly by the group of Bilal El-Zahab [1,2]. The typical Li-O_2 cell Nyquist plot it consists of a low frequency diffusion tail for the porous electrode [3], which can be a constant phase element (CPE) or a finite Warburg element (Z_W). The semicircle can be attributed to the active electrode interface (that also includes the lithium anode [4]) and its diameter is considered to be the charge transfer resistance R_{ct} [31, 37-39]. The high-frequency end of the semicircle is the serial contact resistance of the cell R_S. The low-frequency end can be related to the porous gas diffusion electrode and the reactance is inversely proportional to the electrode capacitance C_{dl} [5].

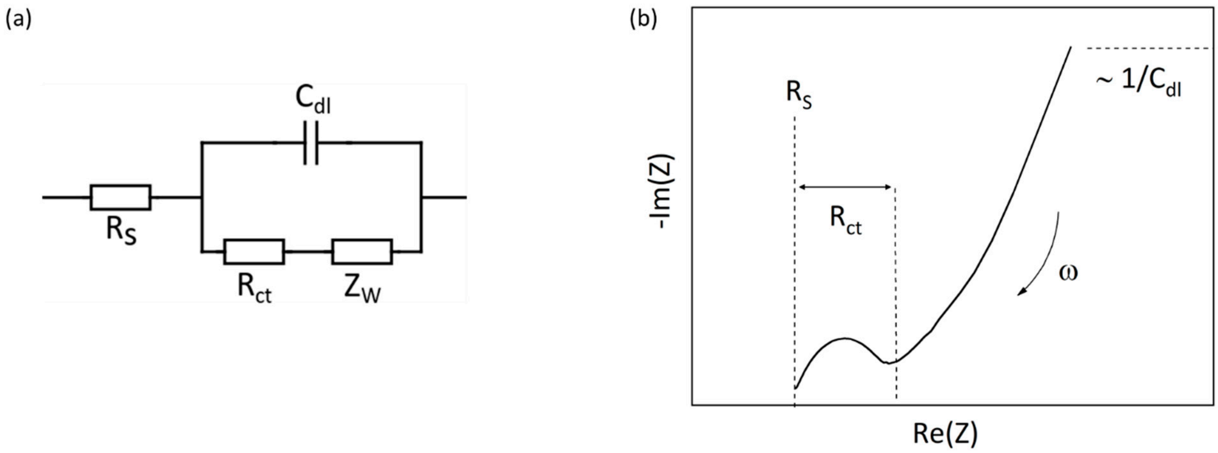

Figure S3. (a) Simple equivalent circuit model of a Li-O_2 battery. (b) Typical Nyquist plot of a Li-O_2 battery (in its equilibrium state prior discharge). Models adapted from literature [1,2].

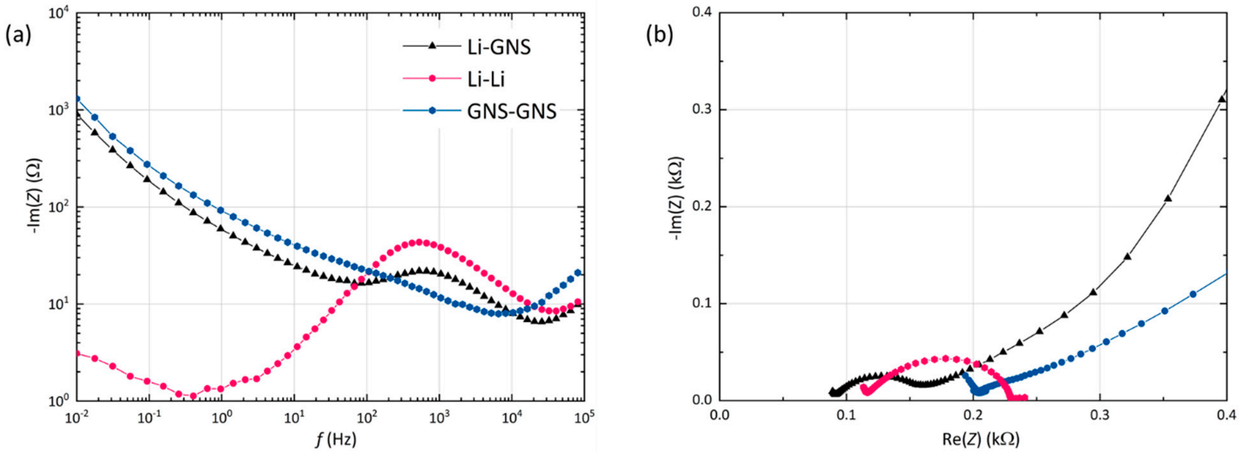

Figure S4. (a) Bode-like and (b) Nyquist plot of a standard full Li-O_2 cell with Li anode and GNS-foam cathode compared to symmetrical cells built with Li-Li or GNS-GNS electrodes. The reactance measured in the Li-Li cell in the frequency region between 100 Hz and 1000 Hz is assigned to the semicircle [4], while the low frequency tail (< 10 Hz) is attributed to the GNS electrode and related Li^+ diffusion processes. The Li-GNS full cells feature both characteristic elements.

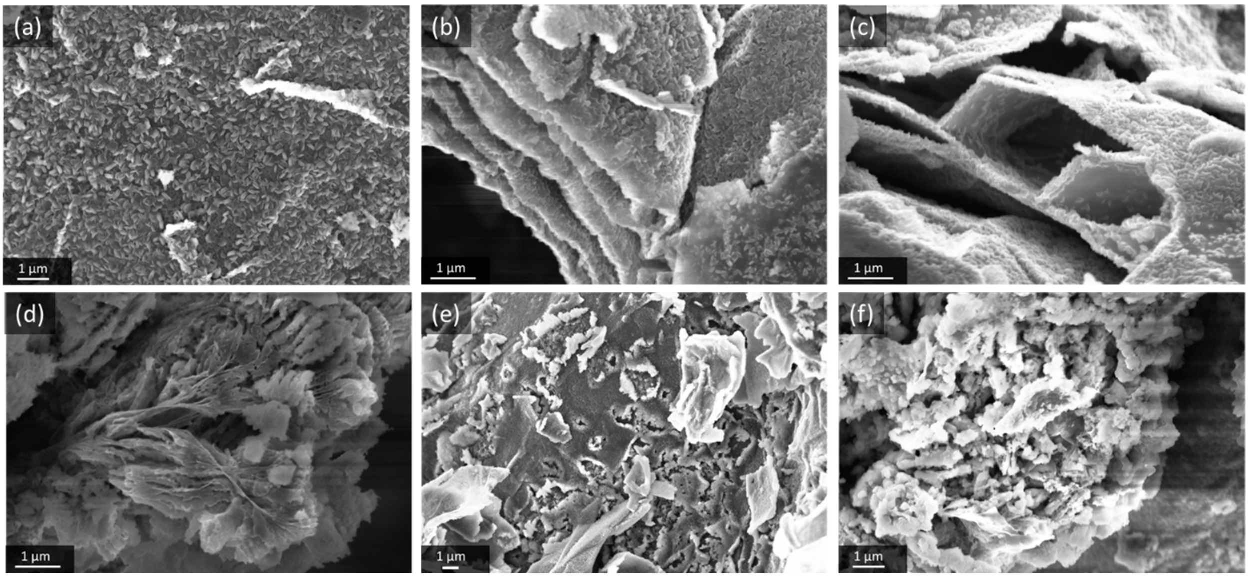

Figure S5. SEM images of damaged GNS after discharge with $LiNO_3$-TEGDME (various discharge conditions). (a) Barely-coated area on the electrode top, previously covered by the current collector disc. (b) Widening of a GNS stack. (c) GNS bending and splaying. (d) Discharge-product-covered GNS splitting at the nanosheet edges. (e) GNS "corrosion" and rupture. (f) Late stage GNS degradation with heavily insulating products that charge up in the electron beam.

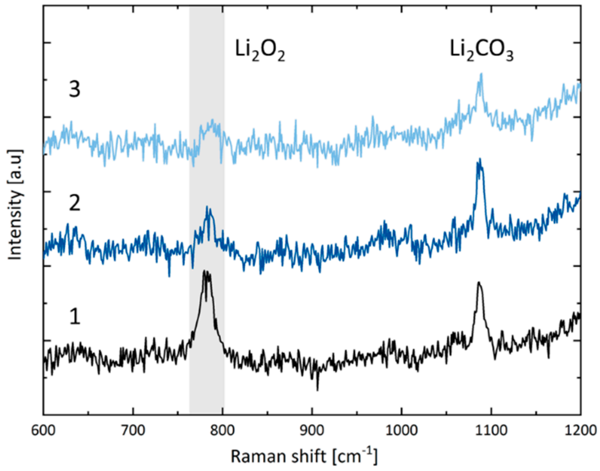

Figure S6. Raman spectrum of a discharge GNS-foam electrode. The fading of the peroxide signal (790 cm^{-1}) is observed for extended laser exposure in three consecutive measurements. The lithium carbonate signal (1095 cm^{-1}) is less affected by the beam-induced decomposition.

References

1. Chamaani, A.; Safa, M.; Chawla, N.; El-Zahab, B. Composite gel polymer electrolyte for improved cyclability in lithium-oxygen batteries. *Acs Appl. Mater. Interfaces* **2017**, *9*, 33819–33826.
2. Chawla, N.; Chamaani, A.; Safa, M.; Herndon, M.; El-Zahab, B. Mechanism of ionic impedance growth for palladium-containing cnt electrodes in lithium-oxygen battery electrodes and its contribution to battery failure. *Batteries* **2019**, *5*, 15–15.

3. Knudsen, K.B.; Vegge, T.; McCloskey, B.D.; Hjelm, J. An Electrochemical impedance spectroscopy study on the effects of the surface- and solution-based mechanisms in li-o2 cells. *J. Electrochem. Soc.* **2016**, *163*, A2065–A2071.
4. Højberg, J.; McCloskey, B.D.; Hjelm, J.; Vegge, T.; Johansen, K.; Norby, P.; Luntz, A.C. an electrochemical impedance spectroscopy investigation of the overpotentials in li-o2 batteries. *Acs Appl. Mater. Interfaces* **2015**, *7*, 4039–4047.
5. Christensen, A.E.; Højberg, J.; Norby, P.; Vegge, T. Impedance-based battery management for metal-o2 systems. *J. Electrochem. Soc.* **2015**, *162*, A2075–A2079.

© 2019 by the authors. Licensee MDPI, Basel, Switzerland. This article is an open access article distributed under the terms and conditions of the Creative Commons Attribution (CC BY) license (http://creativecommons.org/licenses/by/4.0/).
